# Platelet-rich Plasma in Orthopedics: Unraveling Cellular Mechanisms, Therapeutic Potential, and Limitations: Platelet-rich Plasma in Orthopedics

**DOI:** 10.31661/gmj.v14i.3883

**Published:** 2025-11-05

**Authors:** Morteza Nakhaei Amroodi, Khatere Mokhtari, Pouria Tabrizian

**Affiliations:** ^1^ Bone and Joint Reconstruction Research Center, Shafa Orthopedic Hospital, Department of Orthopedic, School of Medicine, Iran University of Medical Sciences, Tehran, Iran; ^2^ Department of Cellular and Molecular Biology and Microbiology, Faculty of Biological Science and Technology, University of Isfahan, Isfahan, Iran

**Keywords:** Platelet-rich Plasma, Orthopedic, Macrophage Polarization, Tendon Repair

## Abstract

The growing interest in autologous biological therapies, such as Platelet-Rich Plasma (PRP), within orthopedic surgery and sports medicine, necessitates refined strategies for post-surgical tissue repair. Despite technological advancements, the proliferation of PRP preparation devices has raised concerns about preparation quality consistency. The absence of consensus on standardization and condition-specific formulations contributes to conflicting outcomes in the literature. Moreover, the potential of personalized treatment protocols, platelet dosage optimization, and PRP’s angiogenic, antimicrobial, and analgesic properties in orthopedic surgery remains underexplored. This review delves into recent advancements in PRP preparation techniques and therapeutic effects, drawing from published data on its applications in orthopedic surgery for tendon injuries, bone repair, spinal fusion, and major joint replacements. Despite promising preclinical study results, clinical trials have shown varying efficacy compared to traditional repair methods. Mechanisms underlying PRP’s actions, including its impact on tendon fibroblasts and macrophage polarization, are scrutinized. While PRP elicits an inflammatory response in tendon fibroblasts, its effect on macrophage polarization remains ambiguous. Additionally, inconclusive findings from studies on PRP’s effectiveness in shoulder surgery underscore the need for standardized protocols and further investigation due to challenges like preparation discrepancies and application techniques. This review focusing on influence on healing quality and pace.

## Introduction

WHAT IS PRP?

**Figure-1:**
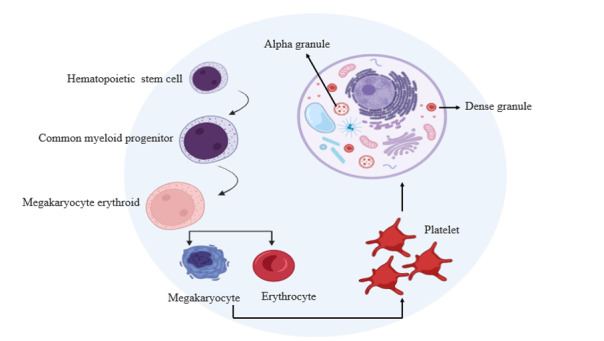
Figure 1. The development and structure of platelets.

In academic literature, Platelet-rich plasma (PRP) has traditionally been characterized as "plasma with a platelet count exceeding the baseline found in whole blood" [1]. The conventional understanding of PRP refers to a concentrated combination of plasma—the cell-free component of blood that contains clotting factors and other bioactive substances critical for wound healing—and platelets, along with their associated growth factors and cytokines. However, the definition of "platelet-rich plasma" has recently expanded to include a variety of derivative formulations (see Table-1). These formulations can differ significantly not only in their platelet concentrations but also in the inclusion of red blood cells and/or white blood cells in the final product. The core method for producing any form of PRP involves plasmapheresis, a process that selectively separates the liquid and cellular components of whole blood [2]. This phenomenon is explained by Stokes' Law, a physical principle stating that the settling velocity of particles in a fluid under the influence of gravity is approximately proportional to their diameter [3][4][5]. Hence, particles possessing greater dimensions, such as red blood cells and white blood cells, will precipitate comparatively quicker than platelets under the influence of gravitational forces. This process enables platelets to sustain suspension primarily within the liquid (plasma) fraction of blood, while larger solid particles like red and white blood cells settle more rapidly, resulting in their separation from platelets due to gravitational effects [6][7][8]. Alpha-granules play a pivotal role in PRP therapy due to their abundance of growth factors, including VEGF, ECGF, IGF-1, PDGF, TGF-β, EGF, PDAF, HGF, FGF, GDNF, PF4, IL-8, and CXCL7. Dense granules, the second-most abundant granules in platelets, store ADP, ATP, calcium, serotonin, and glutamate. Upon PRP treatment, their release contributes significantly to the therapeutic benefits of this approach [9][10]. PRP therapy exhibits a generally favorable safety profile, with only a few absolute contraindications, such as severe thrombocytopenia, platelet dysfunction, unstable hemodynamics, and the presence of sepsis or local infection at the PRP administration site. Relative contraindications encompass recent intake of nonsteroidal anti-inflammatory drugs within 48 hours prior to treatment, glucocorticoid injections within the preceding 2 weeks, recent illness or fever, history of malignancies, anemia with hemoglobin levels below 10 g per deciliter, mild thrombocytopenia, and tobacco usage.[11][12][13] In recent years, there has been a surge of interest in PRP therapy within the medical community, driven by its favorable benefit-to-risk ratio. Approximately 8000 papers have been published on this topic, with over 6000 emerging within the last decade alone, as reported by PubMed. Initially described in hematology during the 1970s for treating patients with thrombocytopenia, PRP gained momentum in the early 1990s due to promising results observed in both monotherapy and combination therapy for a range of medical conditions [14]. There has been a notable expansion in the utilization of PRP within orthopedics, accompanied by promising outcomes. Its application has shown encouraging results in various aspects of musculoskeletal health, including bone fracture healing, injuries to ligaments, muscles, and tendons, treatment of articular cartilage lesions, as well as addressing peripheral nerve injuries. This broad spectrum of applications underscores the versatility and potential effectiveness of PRP in orthopedic care [15] (Figure-1).

## Leukocyte

In LP-PRP, platelets serve as the principal cellular components exhibiting antibacterial activity. In the case of a postoperative infection, they are among the initial responders to detect endothelial injury and the infiltration of microbial pathogens into the bloodstream or tissues. Upon recognition, platelets undergo aggregation and trigger the release of platelet agonists, including ADP, thrombin, and von Willebrand Factor, which collectively promote platelet activation and rapid accumulation at the site of tissue injury [26][27][28] In LP-PRP, in addition to releasing antimicrobial peptides (AMPs), platelets exhibit the capacity to produce reactive oxygen species, adhere to and internalize microorganisms, and participate in antibody-dependent cellular cytotoxicity [29]. LR-PRP buffy coat preparations, in addition to being abundant in platelets, contain a high concentration of viable white blood cells, particularly neutrophils. These immune cells are key components of the innate immune system and play a critical role in protecting the body against infections [30][31]. Prior studies have indicated that oxidative killing, in contrast to nonoxidative mechanisms, constitutes a significant portion of neutrophil's antibacterial effect, with myeloperoxidase (MPO) playing a crucial role in this process [32][33]. PRP has the potential to work synergistically with antibiotics and may serve as an adjunctive therapy for infections, particularly in cases where antibiotic-resistant bacteria are involved, following identification of the pathogen [34]. The impact of leukocytes in PRP on its antibacterial properties is a subject of debate. While leukocytes are vital components of host defense mechanisms, their presence in PRP theoretically should enhance its antibacterial properties. The potential enhancement of antimicrobial properties, especially in LR-PRP, could offer an appealing complement to the established tissue repair and regenerative capabilities of autologous PRP in post-surgical wound healing.

## Neutrophils

Neutrophils play a crucial role as key leukocytes in various healing processes, helping to form dense barriers to defend against invading pathogens. This function is further supported by antimicrobial proteins found within platelets [35][36]. The inclusion of neutrophils is a consideration in defining the objectives of C-PRP treatment. Elevated tissue inflammatory levels may be deemed necessary in PRP biological treatments for chronic wound care or applications aimed at promoting bone growth or healing [37][38]. Significantly, further investigation has revealed additional functions of neutrophils across various models, underscoring their involvement in processes such as angiogenesis and tissue regeneration [30]. Nevertheless, neutrophils can elicit detrimental effects and are therefore contraindicated for certain applications. One study illustrated that the utilization of PRP enriched with neutrophils may lead to an elevated ratio of collagen type III to collagen type I, contributing to fibrosis and reduced tendon strength [39]. Additional detrimental effects mediated by neutrophils include the secretion of inflammatory cytokines and matrix metalloproteinases (MMPs), which contribute to pro-inflammatory and catabolic responses when tissues are exposed to these mediators [40].

## Lymphocytes

In C-PRP, mononuclear T and B lymphocytes are notably enriched compared to other leukocytes. These lymphocytes play a pivotal role in cell-mediated cytotoxic adaptive immunity. They initiate cellular responses to combat infections and adapt to external intruders [41]. Additionally, cytokines produced by T lymphocytes, such as IFN-γ and IL-4, contribute to the enhancement of macrophage polarization [42]. Research findings revealed that conventional T lymphocytes indirectly facilitate tissue healing in a mouse model by influencing the differentiation of monocytes and macrophages [43].

## Monocytes-versatile Cells with Potential for Tissue Regeneration

The presence of monocytes in PRP vials varies depending on the preparation devices employed; however, their inclusion and regenerative potential are seldom addressed in the literature. As a result, monocytes receive limited attention in both preparation protocols and final formulations. These cells constitute a heterogeneous population derived from bone marrow progenitors through hematopoietic stem cell differentiation pathways. Monocytes subsequently migrate to peripheral tissues through the bloodstream in response to microenvironmental signals. During both homeostasis and inflammatory conditions, circulating monocytes leave the vasculature and are recruited to sites of tissue injury or degeneration, where they function as effector cells or differentiate into macrophages [44][45]. In a hypothetical scenario where C-PRP with elevated levels of monocytes is injected into a diseased local microenvironment, it is likely that these monocytes would primarily differentiate into macrophages (MΦs). This differentiation process could trigger substantial cellular changes within the affected area.

## Preparation PRP Formulations

Fadadu and colleagues undertook a comprehensive review of 33 systems and protocols for PRP [46]. One of the key observations indicated that some systems yielded PRP preparations with platelet concentrations below those of whole blood, whereas dual-spin closed systems generated PRP with platelet counts exceeding 1.6 × 10^6/μL. Presently, the clinical characterization of PRP formulations is most accurately based on their absolute platelet concentration, marking a shift from the original definition of PRP, which emphasized achieving levels above baseline. The current standard requires a minimum platelet concentration greater than 1 × 10^6/μL, corresponding to an approximate fivefold increase relative to baseline values [47]. Many contemporary PRP preparation systems have the capacity to produce elevated platelet concentrations and provide diverse formulations of PRP concerning leukocyte and erythrocyte content, as well as concentrations of PGF [48][49].

## Platelet Dosage in PRP Therapies

It's logical to anticipate that PRP formulations with higher platelet concentrations would lead to a more significant release of bioactive factors, potentially affecting outcomes. Multiple studies have suggested that cells react in a dose-dependent manner to PRP. In this regard, Mautner et al. were trailblazers in incorporating the absolute PRP platelet count into a comprehensive PRP classification system [50]. As expected, divergent results concerning optimal platelet dosage have been reported in both clinical trials and in vitro cell culture studies utilizing specific cell types and tissue models [51][52]. One study proposed that a minimum platelet count of 1 × 10^6/µL was necessary for optimal enhancement of bone and soft tissue healing. Similarly, research on PRP in transforaminal lumbar fusion showed a significantly higher fusion rate when the platelet dose surpassed 1.3 × 10^6/µL [53]. A recent report emphasized that achieving a platelet concentration in PRP that is more than five times higher than baseline is crucial for obtaining favorable outcomes in spinal fusion procedures [54]. An in vitro study determined that a platelet dosage of 1.5 × 10^6 platelets/µL was required to trigger tissue repair mechanisms and foster a functional angiogenic response via endothelial cell activity [55]. In addition to dose-dependency, the therapeutic effects of PRP on cellular activity appear to be greatly influenced by the duration of exposure. An in vitro study demonstrated that short-term exposure to human platelet lysates promoted bone cell proliferation and chemotaxis. However, prolonged exposure beyond 48 hours led to a reduction in mineral formation and alkaline phosphatase activity [56]. Tissue culture experiments and numerous clinical studies, especially those focusing on bone growth, have shown increased cell proliferation with PRP treatment, correlating with platelet dosage, especially with platelet counts of at least 1x10^6 /µL [57].

## Clinical use of PRP

**Table:** Table1. Growth Factors, their Origins, and Corresponding Functions are Outlined as Follows

**Growth factor**	**Source**	**Function**	**Ref.s**
	Platelets	neutrophils	macrophages	osteoblasts	mesenchymal cells	endothelial cells	Fibroblasts	other
TGF-b	*	*	*					natural cell killers and cartilaginous matrix	promotes the growth of undifferentiated mesenchymal cells regulates endothelial function	[16][17][18]
FGF	*		*	*	*				mitogen for chondrocytes, osteoblasts	[16][17][18]
PDGF a-b	*		*						Promotes the chemotaxis	[16][17][19]
Epidermic growth factor	*		*						Promotes the mitosis of mesenchymal cells	[16][20][21]
VEGF	*					*			Induces endothelial cell mitosis	[16][21][22]
IGF	*		*	*	*				mitogenesis of mesenchymal cells /triggers osteoblast activity	[16][23][24]
HGF	*				*				Controls cell growth	[25]
KGF					*		*		Controls the migration and proliferation of epithelial cells.	[25]
Ang-1	*	*							Promotes angiogenesis	[25]
PF4	*								Recruits leukocytes	[25]
SDF-1α	*					*	*		Attracts CD34+ cells promoting angiogenesis	[25]
TNF		*						mast cells, T lymphocytes	Controls monocyte migration fibroblast proliferation	[25]

**Table:** Table2. Clinical Trials Examining the Application of PRP in Individuals with Tendinopathies

**Lesion site**	**Outcome**	**Ref.s**
Knee, patellar tendinopathy	improvement	[60]
Knee, patellar tendinosis	improvement	[61]
Knee, patellar tendinopathy	improvement	[62]
Rotator cuff	no significant difference	[63]
Knee, patellar tendinosis	Significant reduction of pain	[64]
Knee, patellar tendinopathy	improvement	[65]
Rotator cuff	no significant difference	[66]
Achilles tendon, chronic tendinopathy	improvement	[67]
Rotator cuff	no significant difference	[68]
Elbow, epicondylitis	improvement	[69]
Rotator cuff	no significant difference	[70]
Elbow, epicondylitis	no significant difference	[71]
Achilles tendon, chronic tendinopathy	without changing the MRI	[72]
Elbow, epicondylitis	improvement	[73]
Achilles tendon, chronic tendinopathy	no significant difference	[74]

**Figure-2:**
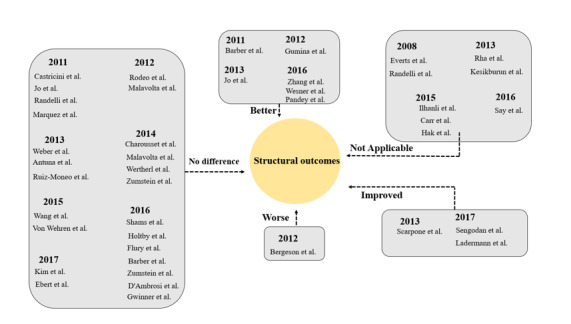
Figure 2. Clinical Studies Evaluating PRP for RCT: Structural Outcomes

**Figure-3:**
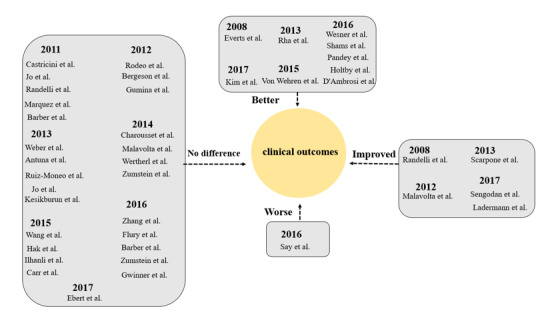
Figure 3. Clinical Studies Evaluating PRP for RCT: clinical outcomes

The clinical use of PRP is based on its ability to enhance the concentration of growth factors and protein secretion, thereby facilitating the cellular healing process. It has been widely employed in the treatment of musculoskeletal injuries to support recovery [58]. Although PRP holds substantial clinical promise, its therapeutic application faces challenges due to a lack of standardized techniques and insufficiently detailed descriptions of the procedures used. As a result, there is an urgent need to establish uniform guidelines for producing high-quality PRP and to conduct further research to identify the optimal platelet concentration for different clinical conditions. Clinical trials on PRP for tendon injuries show considerable variation in preparation methods, quality control, dosage, and injection frequency, making it difficult to assess therapeutic effectiveness. Additionally, differences in the types of cells involved, the release of inflammatory cytokines upon platelet activation, and the varied methods used for PRP activation or non-activation further complicate the analysis [59]. Clinical outcomes resulting from the use of PRP treatments for chronic tendinopathy exhibit a range, spanning from notably positive short- and/or long-term effects to positive outcomes that lack statistical significance. The diversity observed in the stages of chronic tendinopathy could potentially elucidate the differences in outcomes, implying that PRP may be advantageous for specific stages while being less effective for others (Table-2).

## Knee Osteoarthritis

Osteoarthritis stands as the prevailing musculoskeletal disorder, with an estimated prevalence of 10% among individuals aged 60 years and older across the globe [75]. The knee often presents with symptoms, causing pain, disability, and substantial healthcare expenses. Novel biologic and nonoperative treatments, such as intra-articular viscosupplementation and PRP injections, have been suggested for managing the initial phases of osteoarthritis, aiming to alleviate symptoms and postpone surgical procedures. Numerous studies have explored the impact of PRP on knee osteoarthritis, yielding varied outcomes [76][77][78][79]. In 2015, Campbell and associates published a systematic review comprising three overlapping meta-analyses that compared the outcomes of intra-articular PRP injection versus control across 3278 knees [80]. They documented a significant improvement in patient outcome scores for the PRP group compared to the control group from 2 to 12 months following injection. However, due to considerable variability across the studies included, the ideal number of injections or the appropriate intervals between them remains unclear. Meheux and colleagues published a systematic review in 2016 that included six studies (with a total of 817 knees) comparing PRP and hyaluronic acid (HA) injections [81]. Despite variations among studies, most published findings indicate superior symptomatic relief in individuals with initial knee degeneration, implying that PRP utilization could be contemplated for this demographic.

## Frozen Shoulder

Certainly, frozen shoulder is a common condition that leads to significant morbidity [82][83]. Frozen shoulder impacts the glenohumeral (GH) joint, causing limitations in both active and passive movement due to adhesions and fibrosis within the GH capsule, consequently leading to a decrease in joint space [84][85][86][87]. While frozen shoulder typically follows a benign course, with many physicians believing that the condition improves within two or three years, some patients may experience persistent symptoms. According to current knowledge, up to 40% of patients may continue to experience permanent symptoms after three years [88][89]. While corticosteroid and occasionally hyaluronic acid injections may yield positive outcomes for frozen shoulder, some physicians advocate for physical therapy as a treatment option [90][91]. PRP has garnered attention for its application in soft tissue treatment, attributed to its capacity to stimulate collagen production and growth factors, thus augmenting healing by enhancing stem cell activity. Nonetheless, there remains a lack of empirical evidence regarding the efficacy of PRP in treating frozen shoulder. In a case study involving a 45-year-old male with shoulder adhesive capsulitis, the patient underwent two consecutive PRP injections at the seventh- and eighth-month post-symptom onset. Pain levels, function, and range of motion were evaluated using the visual analogue scale, DASH questionnaire, and goniometer, respectively. Following the initial injection, the patient reported a 60% reduction in daytime shoulder pain, absence of nocturnal pain, a twofold enhancement in range of motion, and a greater than 70% improvement in function. This study underscores the potential of PRP in managing frozen shoulder, underscoring the need for further exploration through randomized trials [91].

## Ulnar Collateral Ligament Injuries

The anterior bundle of the ulnar collateral ligament (UCL) plays a pivotal role in stabilizing the elbow against valgus forces. Overhead athletes, particularly those involved in high-velocity throwing sports, are prone to repetitive stress-related injuries to the UCL, which may culminate in partial or complete ligamentous tears. These injuries often manifest as medial elbow pain and can impair both throwing speed and precision. While complete UCL ruptures commonly require surgical reconstruction, there remains a lack of consensus regarding the optimal treatment strategy for partial tears. In recent years, the combined use of platelet-rich plasma (PRP) therapy and structured physical rehabilitation has gained attention as a potential means to enhance recovery in such cases [92]. PRP may be utilized alongside physical therapy and a structured interval throwing regimen for the management of partial UCL tears, with many athletes successfully returning to their pre-injury performance levels. Nonetheless, additional research is warranted to elucidate the precise therapeutic role and efficacy of PRP in this specific athletic population.

## Hamstring Injuries

Acute hamstring injuries are prevalent in various sports, especially those involving sprinting or running. Despite a lack of consensus in the literature regarding the management or definition of return to play (RTP) after hamstring injury, most injuries tend to resolve within 3 to 6 weeks [93]. Significant pain and edema are commonly associated with acute hamstring injuries, particularly at the proximal myotendinous junction of the long head of the biceps femoris and semitendinosus [94][95]. PRP injection near the proximal myotendinous hamstring origin has been proposed as a potential means to expedite the recovery process following acute hamstring injury. However, the current body of literature presents varied and limited evidence concerning the effectiveness of PRP injection therapy for this condition. While certain studies have suggested benefits of PRP therapy compared to standard nonoperative management (which typically includes rest, physical therapy, and nonsteroidal anti-inflammatory drugs) in acute hamstring injury, these findings should be interpreted with caution [96][97]. One study reported that athletes in the PRP group did not exhibit differences in outcome scores compared to controls, but they did return to play earlier [97]. In contrast to these findings, a small case-control study involving NFL players and a retrospective cohort study of athletes with severe hamstring injuries found no difference in return-to-play (RTP) rates between those who received PRP injections and the control group [98][99].

## Tendon Injuries

Tendon injuries and ruptures have emerged as a widespread concern, impacting not only young athletes but also the general population, especially among older individuals. Tendons frequently affected include those surrounding the elbow and wrist, as well as those linked with conditions like patellar and Achilles tendinopathies, and the rotator cuff [100].

## Lateral Epicondylitis

Lateral elbow epicondylitis, commonly known as "tennis elbow," is believed to occur as a result of repetitive wrist extension. It is often observed in individuals with certain comorbidities, such as rotator cuff pathology or a history of smoking [101][102][103][104]. The authors reported a significant 60% improvement in pain scores in patients treated with PRP, compared to a more modest 16% improvement in the control group, 8 weeks post-treatment [105][106]. One study indicated that PRP resulted in a significant reduction in Visual Analogue Scale (VAS) pain scores compared to steroids. However, when PRP was compared to autologous blood, no significant differences were observed [107]. The utilization of PRP has sparked debate in the treatment of lateral epicondylitis. Its effectiveness has been empirically studied and compared to more traditional treatments, leading to ongoing discussion and analysis in the medical community [108][109][105]. In a small case series involving six patients, contrast-enhanced ultrasound imaging was used to demonstrate that PRP injection therapy could induce vascularization at the myotendinous junction of the common extensor tendon for up to six months following the injection [110]. The physiological alterations may occur before noticeable clinical enhancements. Brklijac and co-researchers conducted a prospective observation of 34 patients who continued to suffer from symptoms despite conservative therapy and opted for PRP injections [111]. Randomized controlled trials have indicated no significant difference between PRP and corticosteroid injections in the short-term treatment of symptomatic lateral elbow epicondylitis [112][113]. The current evidence suggests that PRP injection therapy has limited effectiveness in treating lateral epicondylitis, particularly in the short term when compared to corticosteroid injections. However, in the mid to long term, PRP therapy may provide some benefits. Nonetheless, well-designed prospective randomized controlled trials are essential to clarify the effects of PRP in comparison to the natural progression of tendon healing and symptom resolution.

## Patellar Tendon Dysfunction

Patellar tendinopathy, often referred to as jumper's knee, is a common overuse tendon ailment. Platelet-rich plasma shows promise in assisting tissue regeneration, especially in cases with limited healing potential. Despite this promise, there remains a paucity of high-quality evidence regarding the effectiveness of PRP for this condition. However, a recent meta-analysis, encompassing only two randomized controlled trials (RCTs), compared PRP injections with extracorporeal shockwave therapy and dry needling of the tendon. The analysis unveiled no significant difference at the 3-month follow-up, but superior outcomes favoring PRP treatment were noted at longer follow-up periods (6 months or more) [114].

## Achilles Tendinopathy and Rupture

Achilles tendinopathy is a frequent cause of pain in both recreational and competitive athletes [115][116]. Initial conservative management for Achilles tendinopathy typically includes rest, activity and shoe modifications, physical therapy, and eccentric loading exercises. When these strategies fail to relieve symptoms after six months, more invasive treatments may be considered. PRP injection has emerged as an alternative for cases that do not respond to conservative treatment. However, data from several randomized controlled trials indicate that PRP injections do not significantly improve clinical outcomes for Achilles tendinopathy [117]. In a pilot study comparing PRP injections to an eccentric loading program for treating mid-substance Achilles tendinopathy, the results indicated no significant difference in outcomes between the two groups, even though the sample size was small [118]. A study with 54 patients having chronic mid-substance Achilles tendinopathy assessed the impact of eccentric exercise therapy paired with either a PRP injection or a placebo saline injection. Both groups significantly improved in VISA-A scores after 24 weeks, but the differences were not statistically significant. Current research indicates that PRP does not provide additional benefits over conventional treatment for Achilles tendinopathy [119][74]. Nonetheless, recent systematic reviews have underscored the scarcity of high-quality evidence in this domain [120]. Although non-randomized trials have shown promising outcomes, including favorable return to sport participation and sustained benefits lasting up to the midterm, randomized controlled trials have not demonstrated any superiority of PRP over placebo or physiotherapy for Achilles tendinopathy [120]. Specifically, the sole available randomized controlled trial indicated that the incorporation of PRP might potentially impede tissue healing. This is attributed to the absence of biomechanical advantages observed, with PRP patients demonstrating inferior performance compared to the 'suture-alone' group [121]. Future well-designed, prospective randomized controlled trials with larger sample sizes are needed to definitively determine PRP's role in treating Achilles tendinopathy.

## PRP in RC Tears

Rotator cuff tears represent a prevalent source of shoulder discomfort and functional impairment. Their occurrence is on the incline, parallel to the growing engagement of aging individuals in physically demanding activities [122]. Arthroscopic repair has demonstrated favorable outcomes in alleviating pain and improving functional capabilities for individuals with rotator cuff tears [123][124][125][126]. Rotator cuff repair has yielded a significant level of contentment among patients; however, persistent challenges persist, particularly concerning large to massive tears. These difficulties are frequently associated with the inadequate efficacy of treatment, stemming from the complexities involved in reconstructing the tendon-to-bone interface [127]. Many studies have explored the application of PRP during arthroscopic rotator cuff repair (RCR) in an effort to improve and expedite the repair process [66][70][128][129]. However, there is considerable variability among protocols regarding how and when PRP is used to augment the repair. Although basic research literature shows promising findings, the majority of clinical studies utilizing PRP in rotator cuff repair have not exhibited superior outcomes when compared to conventional repair methods. A significant portion of these studies consists of RCTs or, at the very least, comparative studies with control groups. Nevertheless, certain investigations have failed to demonstrate definitive advantageous outcomes of PRP in contrast to a placebo control when it comes to the non-operative treatment of rotator cuff tears [63]. Despite evaluations conducted at multiple time points up to one year, which included assessments such as the Western Ontario Rotator Cuff Index (WORC), Shoulder Pain and Disability Index (SPADI), Visual Analog Scale (VAS) for shoulder pain with the Neer test, and shoulder range of motion, no significant differences in pain or functional outcome scores were observed between the PRP and placebo groups. These findings reflect the varied outcomes reported across studies, underscoring the complexity in determining the efficacy of PRP in the non-surgical treatment of rotator cuff tears. Variability in methodologies and conflicting findings underscore the need for further comprehensive investigations to elucidate the precise clinical effectiveness of PRP in this context. Studies exploring the efficacy of PRP in surgical interventions for various shoulder conditions remain limited. A randomized controlled trial was conducted to assess the efficacy of arthroscopic acromioplasty as a standalone intervention compared to arthroscopic acromioplasty in conjunction with PRP injection for the treatment of rotator cuff tendinopathy.While both intervention groups demonstrated improvements in OSS over the 2-year period following the procedure, no significant difference was observed between the groups. Significantly, shoulders subjected to PRP treatment demonstrated decreased cellularity and vascularity, along with elevated levels of apoptosis, in comparison to those treated solely with arthroscopic acromioplasty [130]. Similarly, a randomized controlled trial compared PRP-augmented arthroscopic needling with unaugmented arthroscopic needling in patients with chronic symptomatic calcific tendonitis. Sub-acromial decompression was performed in 65% of patients in both groups when coracoacromial ligament impingement was present. While both groups showed significant post-operative improvements, no significant differences were found between the two groups at 6 weeks, 3 months, 6 months, and 1 year post-operation in terms of Constant, modified Constant, Quick DASH, or SST scores. Additionally, ultrasound assessments at 3 and 6 months, as well as MRI at 1 year, revealed no notable differences between the groups [131]. As of the current literature review, no articles discussing the use of PRP in the surgical treatment of proximal biceps tendinopathy or labral tears were found. These findings highlight the scarcity of research addressing PRP's effectiveness in surgical interventions for certain shoulder conditions, underscoring the need for further investigation in these areas. Indeed, the study of PRP efficacy in treating shoulder pathology faces several limitations. One critical limitation involves the absence of standardized dosing, formulation, and concentration of platelets and growth factors present in PRP preparations. The inconsistency in defining an optimal PRP composition contributes to the variability in treatment outcomes across different studies [132]. In a single study, there were no significant differences in pre- and postoperative clinical outcome scores between patients who received arthroscopic repair with or without PRP augmentation [132][133]. Overall, most studies have not demonstrated a significant advantage in terms of re-tear rates or shoulder-specific outcomes with the inclusion of PRP during arthroscopic RCR (Figure-2 and -3).

## Macrophages and PRP in Tendon Injuries

As monocytes transition into MΦs, distinct macrophage phenotypes emerge [134]. In the past decade, a model has been developed to clarify the intricate mechanism of macrophage activation, which includes polarization into two distinct phenotypes: MΦ phenotype 1 and MΦ phenotype 2 [135]. The MΦ1 phenotype is characterized by its secretion of inflammatory cytokines such as IFN-γ and production of nitric oxide, contributing to an effective mechanism for combating pathogens. Additionally, the MΦ1 phenotype synthesizes VEGF and FGF. Conversely, the MΦ2 phenotype comprises anti-inflammatory cells that possess an augmented ability for phagocytosis. These MΦ2 cells are responsible for producing extracellular matrix components, angiogenic and chemotactic factors, as well as interleukin-10 (IL-10). In addition to their role in defending against pathogens, MΦ2 cells can dampen the inflammatory response and promote tissue repair. It is noteworthy that the MΦ2 phenotype has been further categorized in vitro into subtypes, such as MΦ2a, MΦ2b, and MΦ2c, based on the specific stimulus encountered [136]. Translating these subtypes into in vivo contexts is challenging, as tissues often harbor mixed populations of MΦs. Intriguingly, pro-inflammatory MΦ1 cells can transition to a pro-repair MΦ2 phenotype in response to local environmental cues and levels of IL-4. Based on these findings, it is reasonable to hypothesize that C-PRP preparations containing elevated concentrations of monocytes and MΦs are likely to facilitate improved tissue repair owing to their anti-inflammatory properties, tissue repair capabilities, and signaling functions [137][138][139][140]. Despite the plethora of clinical outcome studies exploring the impacts of PRP in sports medicine, there continues to be a lack of information concerning its mechanism of action [141][139]. Injured and diseased tendons commonly involve two predominant cell types, fibroblasts, and macrophages, which play a central role in coordinating the healing process [142][143][144]. Fibroblasts play a pivotal role as the primary cells accountable for tendon maintenance and repair, whereas macrophages aid by dismantling damaged tendon tissue. Moreover, macrophages have the capability to release cytokines and other signaling molecules that govern the activity of fibroblasts throughout the healing process [145][146][147][144]. In the initial response to tissue injury, the M1 population of macrophages predominates, engaging in activities such as phagocytosis and apoptosis. Following this, M2 macrophages become the predominant population, directing the repair process and promoting fibroblast proliferation [148][146].

## Effects of PRP Treatment on ECM Remodeling and Macrophage Polarization in Tendon Fibroblasts

Both acute tendon tears and chronic degenerative tendinopathies entail damage or disorganization of the extracellular matrix (ECM), necessitating remodeling and repair by tendon fibroblasts [137][149]. Hyaluronic acid (HA), a glycosaminoglycan, functions as a template for new ECM synthesis. Interestingly, a study revealed that PRP treatment did not exhibit any noticeable impact on the expression of the major HA synthase enzymes, namely HAS1 and HAS2 [150][151]. A study unveiled that PRP treatment resulted in a decrease in the expression of crucial tendinous collagens, specifically collagen 1 and collagen 3, along with elastin, which plays a pivotal role in reestablishing ECM organization post stretching. Additionally, several transcripts responsible for the assembly of mature collagen fibrils were also downregulated subsequent to PRP treatment. These observations align with earlier research on collagen expression and indicate that PRP treatment diminishes the expression of ECM components in tendon fibroblasts [152][153]. Certain transcription factors have been recognized for their significant roles in tendon development, growth, and remodeling. Notably, EGR1, EGR2, and scleraxis are among these transcription factors known to be crucial for tendon biology [154][151], PRP treatment resulted in the downregulation of all three aforementioned genes: EGR1, EGR2, and scleraxis. Furthermore, tenomodulin, which serves as a marker of differentiated fibroblasts, exhibited downregulation in response to PRP treatment [155][156][157]. While TNFα levels are heightened in PRP and can trigger oxidative stress by activating proinflammatory pathways, it is important to highlight that platelets also have the capability to generate and discharge hydrogen peroxide. Consequently, it is conceivable that PRP may contain endogenous peroxides capable of generating reactive oxygen species (ROS), potentially exacerbating oxidative stress in tendon fibroblasts [158]. PRP treatment significantly upregulated the expression of the three enzymes—PTGES, Cox1, and Cox2—implicated in prostaglandin synthesis. However, PRP did not appear to affect the expression of 5-LOX, suggesting that prostaglandins may be involved in PRP-mediated inflammation rather than leukotrienes [159].

Furthermore, alongside the upregulation of PTGES, Cox1, and Cox2, PRP treatment also activated the expression of other proinflammatory transcription factors, including Fosb, Fosl1, and c-Jun. These combined results indicate that PRP treatment significantly and robustly stimulates inflammatory and oxidative stress pathways in tendon fibroblasts [148][142][144].

Different components within PRP have the capacity to polarize cultured macrophages into distinct phenotypes. For instance, IFN-γ and TNF-α can drive macrophages toward an M1 phenotype, while IL-4 and IL-10 are able to promote polarization towards an M2 phenotype [146][160]. PRP treatment resulted in a slight increase in the expression of M1 markers such as iNOS and IL-1β, along with a significant rise in VEGF expression. Conversely, modest increases were noted in the expression of M2a marker Arg1 and M2c markers CD14, IL-10, and CD163. However, with the exception of VEGF, no significant changes were observed in the expression of other macrophage phenotype markers that were evaluated [100]. Therefore, it can be concluded that PRP treatment did not significantly impact macrophage polarization. Notably, despite the considerable change in VEGF expression in macrophages, PRP treatment did not induce a similar change in VEGF expression in tendon fibroblasts. This finding is particularly significant, given that neovascularization is often observed in both acute and chronic tendon disorders [143][144][100].

## Conclusion

In summary, the clinical assessment of PRP formulations lacks consistency, hindering the evaluation of its effectiveness despite technological advancements. The varied composition and lack of standardized dosing affect tissue healing outcomes, contributing to mixed study results. Exploring PRP's potential in diverse formulations and dosing remains underexplored. While standardizing PRP preparation is challenging, adopting uniform platelet dosing for specific conditions could establish quality benchmarks. Calculating total platelet dose is crucial for accurate administration assessment. Well-powered clinical studies are essential for understanding PRP's therapeutic effects fully. Further, the role of leukocytes in PRP efficacy and lack of standardized application techniques pose additional challenges, impeding result comparison and generalization in shoulder pathology treatment with PRP. This study aims to inform orthopedic surgeons about PRP limitations, urging reevaluation for specific conditions.

## Conflict of Interests

There is no conflict of interest.
